# Functionally Mature CD1c^+^ Dendritic Cells Preferentially Accumulate in the Inflammatory Arthritis Synovium

**DOI:** 10.3389/fimmu.2021.745226

**Published:** 2021-10-07

**Authors:** Mary Canavan, Viviana Marzaioli, Vipul Bhargava, Sunil Nagpal, Phil Gallagher, Conor Hurson, Ronan Mullan, Douglas J. Veale, Ursula Fearon

**Affiliations:** ^1^Molecular Rheumatology, Trinity Biomedical Sciences Institute, Trinity College Dublin, Dublin, Ireland; ^2^The European League Against Rheumatism (EULAR) Centre of Excellence for Rheumatology, Centre for Arthritis and Rheumatic Diseases, St. Vincent’s University Hospital, Dublin, Ireland; ^3^Immunology, Janssen Research & Development, Spring House, PA, United States; ^4^Department of Orthopaedics, St. Vincent’s University Hospital, University College Dublin (UCD), Dublin, Ireland; ^5^Department of Rheumatology, Adelaide and Meath Hospital, Dublin, Ireland

**Keywords:** dendritic cells, rheumatoid arthritis, psoriatic arthritis, synovium, maturation

## Abstract

**Objective:**

To examine the role of synovial CD1c^+^DCs in patients with Inflammatory Arthritis (IA) with a specific focus on the transcriptional and maturation signatures that govern their function.

**Methods:**

RNA sequencing was performed on healthy control (HC) peripheral blood (PB), IA PB, and IA synovial fluid (SF) CD1c^+^DCs. Multiparametric flow-cytometry and SPICE analysis were used to examine site [SF and Synovial Tissue (ST) CD1c+DCs] and disease specific characteristics of CD1c^+^DCs, while functional assays such as antigen processing, activation, and MMP production were also performed.

**Results:**

Increased frequency of CD1c^+^DCs (p<0.01) with a concomitant increase in CD80, CCR7 (p<0.01), and CXCR3 (p<0.05) expression was identified in IA PB compared to HC PB. Enrichment of CD1c^+^DCs was identified in IA synovial tissue (ST) (p<0.01) and IA SF (p<0.0001) compared to IA PB, while RNAseq revealed distinct transcriptional variation between PB and SF CD1c^+^DCs. Flow cytometry revealed increased expression of CD83, CD80, PD-L1, and BTLA (all p<0.05) in IA SF CD1c^+^DCs compared to PB, while SPICE identified synovial cells with unique co-expression patterns, expressing multiple DC maturation markers simultaneously. Functionally, synovial CD1c^+^DCs are hyper-responsive to TLR7/8 ligation (p<0.05), have decreased antigen processing capacity (p=0.07), and display dysregulated production of MMPs. Finally, examination of both synovial CD1c^+^DCs and synovial CD141^+^DCs revealed distinct maturation and transcriptomic profiles.

**Conclusion:**

Synovial CD1c^+^DCs accumulate in the inflamed IA synovium in a variety of distinct poly-maturational states, distinguishing them transcriptionally and functionally from CD1c^+^DCs in the periphery and synovial CD141^+^DCs.

## Introduction

Inflammatory arthritis (IA)—which includes rheumatoid arthritis (RA) and psoriatic arthritis (PsA)—are chronic autoimmune diseases primarily affecting the synovial joints. They are characterized by dysregulated angiogenesis, leukocyte infiltration, and lining layer proliferation, leading to the destruction of cartilage and bone (1, 2). A key event in the propagation of synovial inflammation is the infiltration of immune cells including monocytes, lymphocytes, and dendritic cells (DCs), which upon entry to the tissue undergo further activation and differentiation.

DCs are a heterogeneous population of antigen processing cells (APC) that act as sentinels of the immune system, continuously capturing antigens to present to T cells. Functionally, DCs reside *in vivo* in distinct maturation states based on their level of activation. Immature DCs are highly phagocytic, highly migratory, and express low levels of the costimulatory markers CD80, CD86, and CD40. Upon activation, DCs undergo a program of maturation and begin to produce high levels of cytokines, upregulate the expression of costimulatory markers, and express high levels of chemokine receptors such as CCR7 and CXCR3, which facilitate the migration of DCs to inflamed tissues and T cell–rich area of the lymph nodes (3). In humans, DCs can be subdivided into several subsets each differing in ontogeny, location, cytokine production, and immunological functions. Dendritic cells can be broadly classified into two overarching subsets: plasmacytoid DC, which are the main producers of type-I IFN and thus important for antiviral immunity, and the myeloid DC subset (mDCs). mDCs are a more conventional DC subset and thus are the main DC subset responsible for capture, processing, and presentation of antigens on their surface to T cells. mDC can be subdivided into three distinct subsets, each with unique functions: CD141^+^DCs (cDC1), CD1c^+^DCs (cDC2), and the more recently described inflammatory DC3 (4, 5). Functionally, peripheral blood CD141^+^DCs are superior at cross-presentation of exogenous antigens, produce high levels of type III IFNs, and express high levels of TLR3, Clec9A, and XCR-1. However, we previously reported that synovial CD141^+^DCs are also strong inducers of both synovial CD4^+^ and CD8^+^ T cell activation (6). CD1c^+^ DCs are potent activators of CD4^+^ T cells, promoting both Th17 and Th2 immune responses in addition to expressing markers such as CLEC10A and DEC205 (7). Moreover, CD1c^+^DCs express high levels of TLR2, 4–6, 8, and 9 and produce a wide range of cytokines in response to stimulation such as tumor necrosis factor (TNF)-α, IL-1, IL-6, IL-8, IL-12 (8). However, the exploration of DC subsets in human disease has mostly focused on peripheral blood due to the rarity and complexity of phenotyping and isolating DCs from human tissues. Moreover, it is highly likely that DCs play an important role in the pathogenesis of IA due to the strong association of HLA-DR alleles with disease severity in both RA and PsA (9, 10). Furthermore, given the non-redundant role DCs have in naïve T cell activation and the presence of aberrant T cell responses in the inflamed joint, it is likely that DCs contribute to IA disease pathogenesis *via* loss of tolerance resulting in unabated T cell activation.

CD1c^+^DCs have previously been described in the peripheral blood of early RA patients where their diminished frequency is inversely correlated with disease activity (11). A previous study by Moret et al. identified increased frequency of CD1c^+^ DCs in RA synovial fluid (SF) (12); however, the presence of the DCs population has not been explored in RA or PsA synovial tissue to date. Furthermore, while most studies to date have examined peripheral blood CD141^+^ DCs in IA, our previous work first described enrichment of CD141^+^DCs at the site of inflammation, demonstrating distinct transcriptional signatures and functional capacity compared to their blood counterparts (6). This suggests that while DCs activation and function may differ in the periphery, this response is potentiated at the site of inflammation. However, limited studies exist exploring CD1c^+^DCs in IA synovial tissue and SF or indeed DC maturation, which is essential to their function.

While previous histological studies have identified pan-DC markers such as CD11c and CD123 in the IA synovium (13–15), the promiscuous expression of these markers on other populations has led to the increased requirement for the use of in-depth multicolor flow cytometry to identify specific DC subsets. Furthermore, with the emergence of high-throughput technologies such as RNA sequencing, the ability to explore unique tissue-specific DC populations and their maturation capabilities in detail can be achieved. Therefore, the aim of this study was to use in-depth functional and transcriptomic analysis to explore the role of CD1c^+^DCs within the IA synovium to identify disease and site-specific attributes of IA synovial CD1c^+^DCs. Using multicolor flow cytometry in addition to a computational system biology approach, we aimed to identify key signaling pathways and unique maturation phenotypes distinct to synovial CD1c^+^DCs that may provide insight into novel site-specific therapeutics. Finally, we performed comparative analysis of synovial CD1c^+^DCs and CD141^+^DCs, demonstrating distinct signaling pathways and unique maturation phenotypes that distinguish these synovial mDC subsets.

## Materials and Methods

### Patient Recruitment, Arthroscopies, and Sample Collection

Patients with active Rheumatoid Arthritis and Psoriatic Arthritis were recruited from outpatient clinics at the Department of Rheumatology, St. Vincent’s University Hospital and Tallaght University Hospital. Arthroscopy of the inflamed knee was performed under local anesthetic, using a 2.7 mm needle arthroscope (Richard Wolf, IL, USA). Biopsies were utilized to establish whole tissue synovial cell suspensions (16). Peripheral blood and synovial fluid mononuclear cells were also obtained at arthroscopy or Rheumatology Clinics. Patient demographics are included in the table below. Healthy control synovial tissues were obtained at the time of anterior cruciate ligament surgery (ACL) and were used to establish whole tissue synovial cell suspensions. Patient demographics, including gender, diagnosis, treatments, synovitis, and DAS28, are described in **Supplementary Table 1**.

### Study Approval

Ethical approval to conduct this study was granted by St. Vincent’s Healthcare Group Medical Research and Ethics Committee and the Tallaght Hospital/St. James’s Hospital Joint Research Ethics Committee, and all patients and healthy subjects gave fully informed written consent prior to inclusion.

### Cell Isolation

Synovial tissue biopsies were mechanically and enzymatically digested using the GentleMacs system (Miltenyi Biotech) using a soft tumor dissociation kit (Miltenyi Biotech), according to the manufacturer’s instructions. Briefly, synovial biopsies were placed in 4.7 ml of RPMI supplemented with 200 μl of enzyme H, 100 μl of enzyme R, and 25 μ enzyme A in a gentleMACS C Tube followed by initial mechanical disruption of the tissue on a gentleMACS Dissociator. Samples were then incubated for a total of 30 min at 37°C under continuous rotation. A single synovial cell suspension was generated and filtered through a 70 μm cell strainer. In addition, peripheral blood mononuclear cells (PBMC) and SF mononuclear cells (SFMC) were isolated by density gradient centrifugation (Lymphoprep, Stemcell Technologies) for direct comparison of DCs in the circulation *versus* the inflamed synovium. Samples were stained with a panel of fluorochrome-conjugated antibodies for flow cytometry as described in **Supplementary Table 2**. CD1c^+^DCs or CD141^+^DCs were magnetically sorted from total SFMC or PBMC using CD1c or CD141 conjugated magnetic beads (Miltenyi Biotech) according to the manufacturer’s protocols.

### Immunophenotyping and Flow Cytometry

Single-cell suspensions were obtained as described above and used for (1) immunophenotyping or (2) functional experiments. For flow cytometry functional experiments and immunophenotyping experiments, cells were stained as follows: Single-cell suspensions were washed in PBS and incubated with LIVE/DEAD Fixable Near Infrared (Thermo Fisher Scientific) viability reagent as per the manufacturer’s instructions. Cells were then incubated with TruStain FcX receptor blocking solution (BioLegend) prior to antibody staining to minimize non-specific binding. Cells were subsequently stained with a panel of fluorochrome-conjugated antibodies to characterize DC subsets and activation for 30 min at 4°C (**Supplementary Table 2**). Following incubation, cells were washed twice in FACS buffer (PBS with 2% FBS and 0.002% w/v sodium azide), fixed in 1% PFA before being acquired on a four-laser LSRFortessa cytometer (BD). To adjust for spectral overlap between detectors, compensation was applied using single-stained compensation beads (BD Biosciences). For DC activation experiments, 100,000 sorted DCs were stimulated with 1 µg/ml R848 (Invitrogen) for 18 h in complete media after which the cells were harvested and the expression of costimulatory markers was analyzed by flow cytometry as described above. Acquired samples were subsequently analyzed using Flowjo software (Treestar Inc.), and Fluorescence Minus One (FMO) controls were used to determine positivity and gating boundaries. Gating strategies to identify CD1c^+^DCs are depicted in **Supplementary Figure 1**. Frequencies of DCs are represented as percentage of CD1c^+^DCs in the mDC gate. To examine co-expression patterns of costimulatory markers on cells, SPICE (Version 5.1) was used. SPICE, or Simplified Presentation of Incredibly Complex Evaluations, is a data-mining software that enables the analysis of large datasets from polychromatic flow cytometry and organizes the normalized data graphically. A Boolean gating strategy was adopted to identify all possible cell populations and imported into the SPICE program. Pie charts generated as a result of this analysis represent frequency of enzyme co-expression of costimulatory markers whereby single costimulatory marker–positive cells are represented by individual arcs surrounding the pie charts, while double, triple, and quadruple costimulatory marker–positive cells were represented by overlapping arcs (17).

### Multiplex ELISA

CD1c^+^DCs were isolated from PBMC/SFMC, and 100,000 cells were cultured overnight in 100 µl complete media—Dulbecco’s RPMI 1640 (Thermo Fisher Scientific) supplemented with 10% HyClone™ FBS (Sigma), and 1,000 U/ml penicillin streptomycin (Sigma). Supernatants were then harvested and used to measure MMP-1, MMP-9, MMP-3 by multiplex ELISA (Meso Scale Discovery) as per the manufacturer’s instructions. Briefly, 25 µl of supernatants was added to precoated multiplex wells for 2 h at RT under constant shaking. Simultaneously, an eight-point standard curve was added to the plate with a lower limit of detection of 11 pg/ml (MMP-1), 2.1 pg/ml (MMP-3), and 99 pg/ml (MMP-9). The plate was subsequently washed three times using PBS/Tween, after which 25 µl of Detection antibody was added for 2 h at RT under constant shaking. Finally, after washing three times, 150 µl of Read buffer was added, and the plate was analyzed using a Meso Sector S600 Imager.

### Antigen Uptake and Processing Assay

CD1c^+^DCs were isolated from PBMC/SFMC as described above, washed, and 100,000 cells were subsequently incubated with DQ Ovalbumin (Invitrogen) (25 µg/ml) in complete media for 15 min at 4°C and 37°C. Cells were centrifuged at 1,200 rpm for 5 min, the supernatant was removed, and cells were washed using cold PBS. This step was repeated twice before the samples were immediately read on the LSRFortessa flow cytometer. Cells were excited with the 488 nm laser, and fluorescence was detected using the 530/30 bandpass filter. Positive gates were set using the 4°C sample as a control.

### RNA Sequencing

Total RNA was extracted from CD1c^+^DCs IA SF, IA peripheral blood, HC peripheral blood, and CD141^+^ DCs IA SF. RNA samples were reverse transcribed and sequencing libraries constructed using NuGen Ovation Universal RNA-Seq System (CAT# 0402-A01) according to the manufacturer’s protocol. The resulting sequencing libraries were analyzed using the Caliper LabChip GX and quantified using KAPA qPCR. Libraries were then normalized and pooled in one batch of six and one batch of five. Each pool was clustered and sequenced on an Illumina NextSeq500 instrument using 2×100 bp paired-end reads, following the manufacturer’s protocols. The average number of reads per sample was 117 million reads with a minimum of 90.1 million reads. Raw read quality was evaluated using FastQC, and statistical analysis was performed as previously described (6, 18). Data are available on NCBI GEO with the accession number GSE151897 and GSE108174.

### Statistical Analysis

GraphPad Prism 8 software was used for statistical analysis. Mann Whitney test was used for analysis of non-parametric data. P values of less than 0.05 (*p<0.05) were determined as statistically significant.

## Results

### Enhanced Maturation and Migratory Capacity of Circulating CD1c^+^DCs in IA

**Figure 1:**
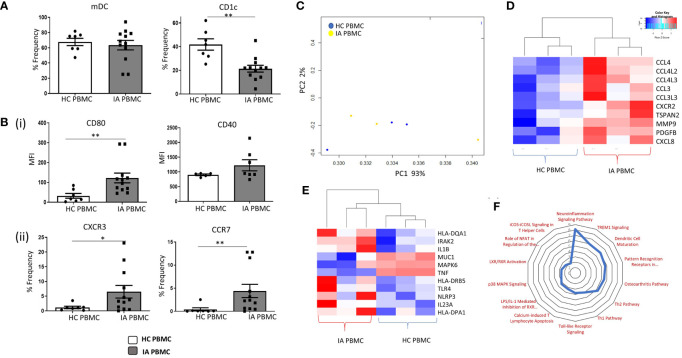
Enhanced Maturation and Migratory Capacity of Circulating CD1c+ DCs in IA. **(A)** The percentage frequency of mDCs and CD1c^+^ DCs in HC peripheral blood (n = 7) and IA peripheral blood (n = 12). mDCs were gated as Lineage- HLADR^+^ CD11c^+^, while CD1c^+^ DCs were gated based on the expression of CD1c within the mDC population. **(Bi)** Median Fluorescence Intensity (MFI) of CD80 and CD40 on CD1c^+^ DCs from IA (n = 7–12) and HC peripheral blood (n = 5–7). **(Bii)** Percentage frequency of CXCR3^+^ and CCR7^+^ CD1c^+^DCs in IA (n = 12) and HC peripheral blood (n = 7). **(C)** RNA sequencing isolated CD1c^+^ DCs from HC (n=3) and IA peripheral blood (n = 3). PCA of total datasets from HC and IA peripheral blood. **(D)** Clustered heatmap representing DEG involved in DC Maturation and **(E)** migration. **(F)** Radar plot representing enriched pathways in IA peripheral blood CD1c^+^DCs compared to HC using IPA. Non-parametric unpaired Mann Whitney U test was used. *p < 0.05, **p < 0.01 indicate groups that are significantly different from each other.

**Figure 2:**
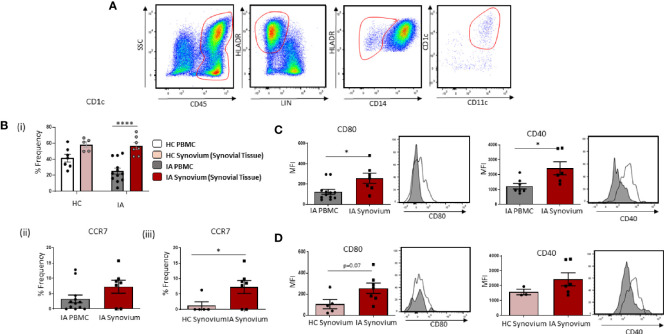
Accumulation of Mature CD1c^+^ DCs in IA Synovial Tissue. **(A)** Gating strategy to identify CD1c^+^ DCs in IA synovial tissue. Following the elimination of debris, doublets, and dead cells, cells were gated as CD45^+^ Lineage ^–^ HLADR^+^ CD14^−^ CD11c^+^ CD1c^+^. **(Bi)** The percentage frequency of CD1c^+^ DCs in HC PBMC (n = 7), HC synovial tissue (n = 5), IA PBMC (n = 12), and IA synovial tissue (n = 7). CD1c^+^DC frequency is represented as a percentage of cells in the mDC gate. **(Bii)** The percentage frequency of CCR7^+^ CD1c^+^ DCs in IA PBMC (n = 12) and IA synovial tissue (n = 7). **(Biii)** The percentage frequency of CCR7^+^ CD1c^+^ DCs in HC synovial tissue (n = 5) and IA synovial tissue (n = 7). **(C)** MFI of CD80 and CD40 expression on CD1c^+^DCs from IA PBMC (n = 7–12) and IA synovial tissue (n = 6–7). **(D)** MFI of CD80 and CD40 expression on CD1c^+^DCs from HC synovial tissue (n = 3–5) and IA synovial tissue (n = 6–7). Non-parametric unpaired Mann Whitney test was used. Where multiple comparisons were determined, two-way ANOVA was used. *p < 0.05, **p < 0.01, ****p < 0.0001 indicate groups that are significantly different from each other.

The frequency of mDCs and specifically CD1c^+^DCs was assessed in IA and HC peripheral blood. mDCs were identified as Lin^–^HLA-DR^+^CD11c^+^ cells. CD1c^+^cells were subsequently identified within this mDC gate based on the positive expression of the CD1c marker. While no differences were observed in the mDCs population between HC and IA PBMC, we identified a significant decrease in the percentage of circulating CD1c^+^DCs in IA patients compared with HC (**Figure 1A**; p<0.01). Furthermore, a significant increase in the expression of CD80 on CD1c^+^ DCs and a trending increase in CD40 in IA patients were demonstrated (**Figure 1B** top; p<0.01). We also identified a significant increase in the frequency of the chemokine receptors CXCR3 and CCR7 in IA peripheral blood compared to HC (**Figure 2B** bottom; p<0.05 and p<0.01 respectively). We also performed RNAseq analysis of both HC peripheral blood CD1c^+^DCs and IA peripheral blood CD1c^+^DCs. Using hierarchical clustering and Principal Component Analysis (PCA), we demonstrated clustering of both peripheral blood populations together, indicative that the global transcriptomes of both IA and HC peripheral blood CD1c^+^DCs are similar (**Figure 1C**). However, following hierarchical clustering analysis of differentially expressed genes (DEG) between IA PB and HC PB, we noted distinct separation of gene clusters between the two groups, suggesting that specific transcriptional characteristics differentiate CD1c^+^DCs from IA PB compared to HC PB (**Supplementary Figure 2**). Upon further examination of IA PB and HC PB, we attributed this separation to a large number of DEGs involved in DC maturation (**Figure 1D**) and DC migration (**Figure 1E**) differentially expressed in IA PB compared to HC PB CD1c^+^DCs. Moreover, ingenuity pathway analysis (IPA) revealed several signaling pathways that were enriched in IA peripheral blood compared to HC (**Figure 1F**). Notably, we demonstrate an enrichment in the DC maturation pathway in IA peripheral blood CD1c^+^DCs (**Figure 1F**), concurring with our *in-vitro* flow cytometry analysis in **Figures 1A, B**.

### Accumulation of Mature CD1c^+^DCs in IA Synovial Tissue

Given the reported decrease in circulating CD1c^+^DCs in IA, with increased expression of chemokine receptors and migratory genes, we hypothesized that peripheral blood CD1c^+^DCs migrate and accumulate in the inflamed synovium. In support of this hypothesis, we sought to investigate the frequency of cells in the circulation *vs* the site of inflammation. Gating strategy is shown in **Figure 2A**. We demonstrate a significant enrichment of CD1c^+^DCs within the tissue compared to IA peripheral blood (**Figure 2Bi**; p<0.0001), whereas there is no significant change in the frequency of CD1c^+^DCs in HC synovium compared to HC peripheral blood (**Figure 2Bi**). Interestingly, however, a similar percentage frequency of CD1c+DCs was observed in HC synovial tissue compared to IA synovial tissue. However, the overall number of cells isolated from HC tissue following digestion was considerably lower than that of IA synovial tissue (~200,000 cells compared to ~3–4 million, respectively), indicative of the increased inflammatory burden observed within the IA synovium. While not examined within this study, this suggests that the absolute counts of CD1c^+^DCs may be lower in the healthy synovium, despite the similar percentage frequency between IA and HC synovial tissue. Moreover, our data, as highlighted below, also suggest that while CD1c+DCs are present in the healthy synovium, they reside there in a less mature state compared to IA synovial tissue. We also report a significant increase in the frequency of CCR7 on IA synovial CD1c^+^DCs when compared to IA peripheral CD1c^+^DCs (**Figure 2Bii**) and HC synovial CD1c^+^DCs (**Figure 2Biii**; p<0.01). In parallel, IA synovial CD1c^+^DCs are more mature with significantly increased expression of CD80 and CD40 compared to peripheral blood CD1c^+^DCs (**Figure 2C**; both p<0.01). Moreover, an increase in CD80 (p=0.07) on IA DCs compared to HC was also observed (**Figure 2D**).

### Distinct Transcriptomic Variation in Synovial CD1c^+^DCs in IA

**Figure 3:**
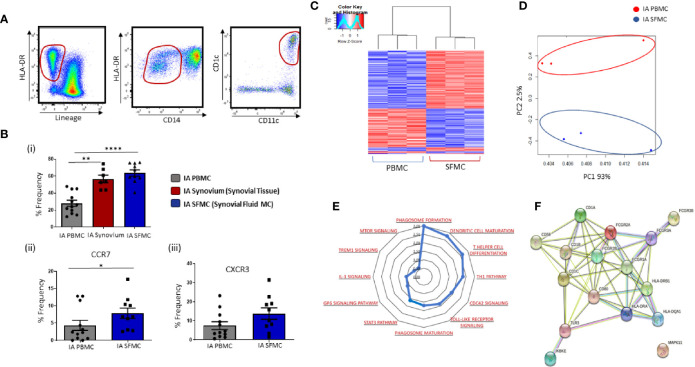
Distinct Transcriptomic Variation in Synovial CD1c+ DCs in IA. **(A)** Gating strategy to identify CD1c^+^ DCs in IA SFMC. Following the elimination of debris, doublets, and dead cells, cells were gated as Lineage ^–^ HLADR^+^ CD14^−^ CD11c^+^ CD1c^+^. **(Bi)** Comparative percentage frequency of CD1c^+^ DCs in IA PBMC (n=12), IA synovial tissue (n = 7), and IA SFMC (n = 7). **(Bii, iii)** The percentage frequency of CCR7^+^ CD1c^+^ DCs and CXCR3^+^ CD1c^+^ DCs in IA PBMC (n = 12) and IA SFMC (n = 10). CD1c^+^DC frequency is represented as a percentage of cells in the mDC gate. **(C)** Hierarchical clustering analysis following RNAseq on DEG from CD1c^+^ DCs IA PBMC (n = 3) and IA SFMC (n = 3). **(D)** PCA of total datasets from CD1c^+^ DCs from IA PBMC and IA SFMC. **(E)** Radar plot representing enriched pathways in CD1c^+^ DCs from IA SFMC CD1c+DCs compared to HC using IPA. **(F)** STRING pathway analysis of specific DC maturation pathway gene signature. Non-parametric unpaired Mann Whitney test was used. Where multiple comparisons were determined, two-way ANOVA was used. *p < 0.05, **p < 0.01, ****p < 0.0001 indicate groups that are significantly different from each other.

Gating strategy to identify CD1c^+^ DCs in IA SFMC is shown in **Figure 3A**. Analysis of both synovial tissue and fluid demonstrated that CD1c^+^DCs were enriched to a similar degree in both synovial compartments compared to HC (**Figure 3Bi**; p<0.001; p<0.0001, respectively). Moreover, SF CD1c^+^DCs have increased expression of the chemokine receptors CCR7 (p<0.05) (**Figure 3Bii**) and CXCR3 (**Figure 3Biii**), indicative of increased migration to the joint. Therefore, for the remainder of the study, we focused on the analysis of CD1c^+^DCs within the SF, which is an easier biological sample to perform downstream functional analyses. RNAseq was performed, and using hierarchical clustering and PCA, we demonstrated that synovial CD1c+DCs clustered separately from IA PB CD1c^+^DCs and presented a distinct transcriptional signature between SF and PB CD1c^+^DCs (**Figures 3C, D**).

To examine site-specific signaling pathways of CD1c^+^DCs, IPA was performed on SF and peripheral blood CD1c^+^DCs. This analysis revealed enrichment in several key inflammatory pathways that are over-represented in IA synovial CD1c^+^DCs compared to IA peripheral blood DCs (**Figure 3E**), including TLR, mTOR, and IL-1 signaling in addition to DC maturation. Moreover, several genes associated with DC maturation were differentially expressed in IA synovial CD1c^+^DCs compared to IA peripheral blood such as CD80, CD58, HLA-DRA with known and predicted interactions depicted in **Figure 3F** using STRING analysis.

### Unique Costimulatory Marker Co-Expression on Synovial CD1c^+^DCs

**Figure 4:**
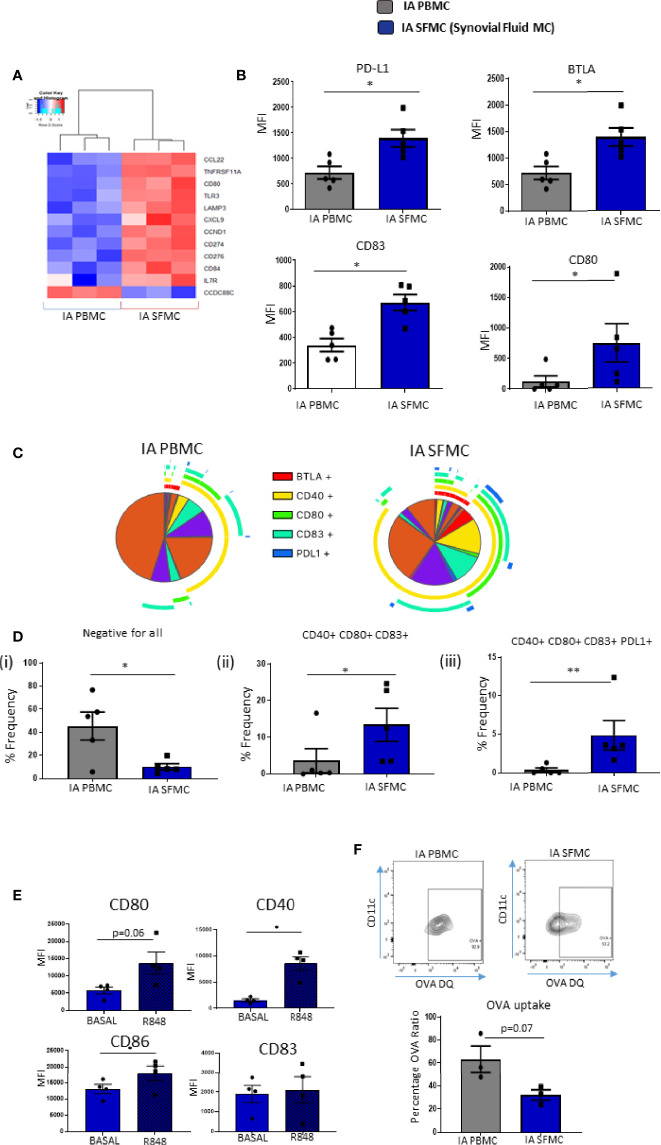
Unique Costimulatory Marker Co-Expression on Synovial CD1c^+^ DCs. **(A)** Clustered heatmap representing DEG involved in DC maturation from CD1c^+^ DCs in IA PBMC and IA SFMC. **(B)** MFI of CD83, CD80, PD-L1, and BTLA on CD1c^+^ DCs from IA (n = 5) and IA SFMC (n = 5). **(C)** SPICE analysis charts demonstrating the co-expression profile of DC maturation markers on CD1c^+^ DCs from IA PBMC and IA SFMC. Each section of the pie chart indicates different combinations of maturation marker. The pie chart arcs indicate the maturation markers expressed by each proportion of cells. **(D)** Percentage frequency of the most differentially distinct co-expression patterns from the SPICE analysis on CD1c+DCs from IA PBMC (n = 5) and IA SFMC (n = 5). **(E)** MFI of CD80, CD40, CD86, and CD83 expression on SF CD1c^+^ DC unstimulated or stimulated for 18 h with R848 (1 µg/ml) (n = 4). **(F)** Percentage frequency of OVA-positive CD1c^+^ DCs from IA PBMC and IA SFMC (n = 3) and representative flow cytometry dot plots. Non-parametric unpaired Mann Whitney test was used. Where multiple comparisons were determined, two-way ANOVA was used. *p < 0.05, **p < 0.01 indicate groups that are significantly different from each other.

Given the presence of increased maturation markers on synovial tissue CD1c^+^DCs, and the emergence of DC maturation as an enriched pathway within SF CD1c^+^ DCs, we next performed further analysis on DC-specific maturation and costimulatory markers in SF and peripheral blood DCs. We identified several costimulatory and coinhibitory genes that were dysregulated in synovial *vs* peripheral CD1c^+^DCs (**Figure 4A**). Furthermore, we demonstrated a significant increase in the expression of CD83, CD80, PD-L1, and BTLA (all p<0.05; **Figure 4B**) in IA SF CD1c^+^DCs compared to peripheral blood DCs. Using a Boolean gating strategy and SPICE analysis, we demonstrated striking differences in synovial CD1c^+^DC maturation markers compared with circulating CD1c^+^DCs (**Figure 4C**). Single-positive cells are represented by individual arcs surrounding the pie charts, while double-, triple-, quadruple-, and quintuple-positive CD1c^+^DCs are represented by overlapping arcs (**Figure 4C**). These data suggest that the maturation profile in SF DCs is more complex than peripheral DCs, with the emergence of synovial cells, which express unique co-expression patterns of multiple DC maturation markers and thus represent DCs in a variety of poly-maturational states. We subsequently extrapolated the most differentially distinct co-expression patterns and demonstrated a decrease in CD1c^+^DCs that were negative for all five markers in IA SFMC *vs* IA PBMC (p<0.05; **Figure 4Di**), confirming an increase in co-expression of maturation markers in the IA SF CD1c^+^DCs. Aligned with this, we observed a significant increase in the presence of CD40^+^CD80^+^CD83^+^ and CD40^+^CD80^+^CD83^+^PD-L1^+^ cells in IA SF compared to peripheral blood (**Figures 4Dii, iii**; p<0.01 and p<0.05, respectively). These data suggest that CD1c^+^DCs in the synovium are more mature and have unique maturation co-expression patterns that are absent from their peripheral blood counterparts.

### Synovial CD1c^+^DCs Are Functionally Mature and Have Decreased Phagocytic Capabilities

We demonstrated that synovial CD1c^+^DCs have a mature phenotype; therefore, we next determined whether mature synovial CD1c^+^ DCs were tolerogenic or still capable of further maturation in response to TLR7/8 ligands in the joint. While synovial CD1c^+^DCs express high levels of CD80, CD83, CD86, and CD40 at baseline, following stimulation with the TLR7/8 ligand, there was a significant increase in CD40, CD86 (both p<0.05; **Figure 4E**) and a trending increase in CD80 (p=0.06) in response to R848 (**Figure 4E**). In parallel, we report a trending decrease in the ability of SF CD1c^+^DCs to process antigen compared to peripheral blood (**Figure 4F**; p=0.07). While not statistically significant, further analysis is required to confirm this observation. In addition, synovial CD1c^+^DCs have reduced production of the matrix metalloproteinases MMP-1 and MMP-9 with increased secretion of MMP-3 compared to peripheral blood CD1c^+^DCs (**Supplementary Figure 3**). Collectively, these data suggest that synovial CD1c^+^DCs are immunogenic and are functionally mature within the synovium.

### Synovial CD1c^+^DCs and Synovial CD141^+^DCs Have Differential Costimulatory Marker Expression

**Figure 5:**
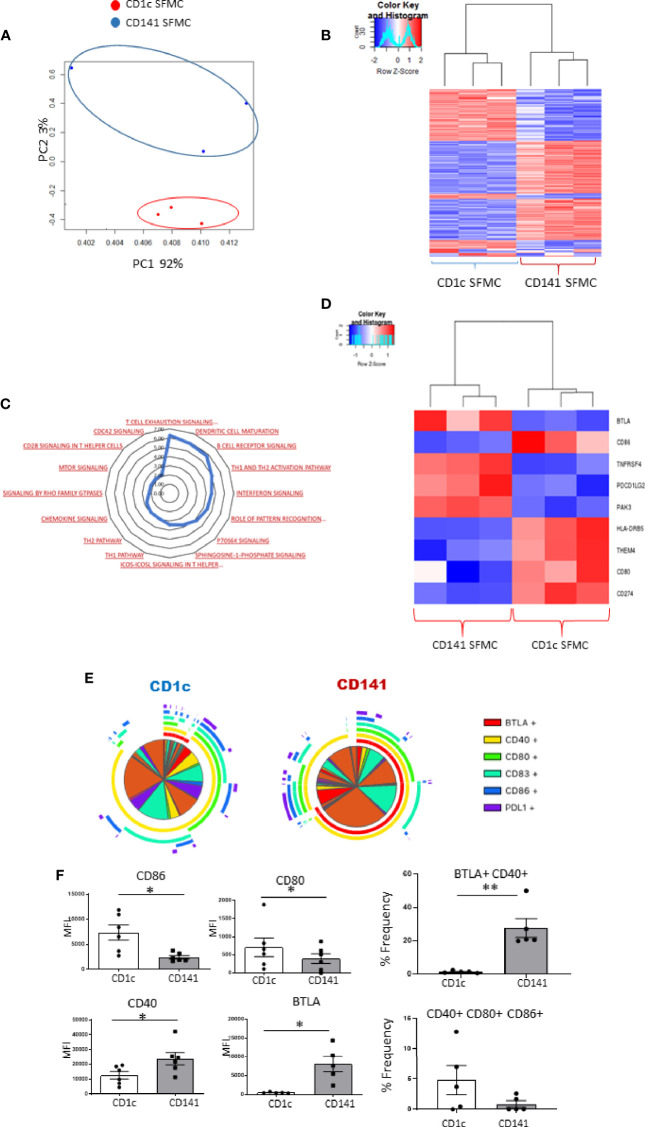
Synovial CD1c+ DCs and Synovial CD141^+^ DCs have differential costimulatory marker expression: **(A)** RNA sequencing was performed on SF CD1c^+^ DCs and SF CD141^+^DCs (n = 3). PCA of total datasets from SF CD1c+ DCs and SF CD141^+^ DCs. Hierarchical clustering analysis on DEG from SF CD1c^+^ DCs and SF CD141^+^DCs. **(B)** Hierarchical clustering on the DEGs from SF CD1c^+^ DCs and SF CD141^+^DCs. **(C)** Radar plot representing enriched pathways in SF CD1c^+^ DCs compared to SF CD141^+^ DCs using IPA. **(D)** Clustered heatmap representing DEGs involved in DC maturation from SF CD1c^+^ DCs and SF CD141^+^ DCs. **(E, F)** Heatmap coded pie charts demonstrating the co-expression profile of DC maturation markers on SF CD1c^+^ DCs and SF CD141^+^ DCs. Each section of the pie chart indicates different combinations of maturation marker expression. The pie chart arcs indicate the maturation markers expressed by each proportion of cells. **(F)** MFI of CD86, CD80, CD83, CD40, BTLA, and PD-L1 on SF CD1c^+^ DCs and SF CD141^+^ DCs (n = 5). Percentage frequency of the most differentially distinct co-expression patterns from the SPICE analysis on SF CD1c+ DCs and SF CD141^+^ DCs (n = 5). Non-parametric unpaired Mann Whitney test was used. *p < 0.05 indicates groups that are significantly different from each other.

We previously reported the first description of mature CD141^+^DCs in IA SF (6), and in this study, we identified an enriched population of mature synovial CD1c^+^DCs. Therefore, we next examined a previously unexplored comparison of both mDC populations in the IA synovium using RNAseq analysis. PCA demonstrated that synovial CD1c^+^DCs clustered separately from synovial CD141^+^DCs, indicating distinct transcriptional variation between both synovial DC subtypes (**Figure 5A**). Furthermore, unsupervised hierarchical clustering on the DEGs also demonstrated that IA SF CD1c^+^DCs clustered separately from synovial CD141^+^DCs (**Figure 5B**). This suggests that although both DC populations are classed within the mDC family and are recruited and enriched in the IA synovium, distinct transcriptional profiles may govern their function. IPA identified enrichment of several signaling pathways in the CD1c^+^DC population compared to the CD141^+^DC population (**Figure 5C**), including mTOR, chemokine, and interferon signaling. Interestingly, DEGs associated with DC maturation were also differentially expressed between the two synovial subtypes (**Figure 5D**). Furthermore, SPICE analysis revealed striking differences in the single and combined expression of DC maturation markers (**Figure 5E**), with synovial CD1c^+^DCs displaying significantly higher expression of CD80 and CD86 compared to CD141^+^DCs (**Figure 5F**; both p<0.05), while synovial CD141^+^DCs have significantly higher levels of CD40 and BTLA compared to the CD1c population (**Figure 5F**; both p<0.05). In addition, a significant increase in the frequency of the BTLA^+^CD40^+^ double-positive population was demonstrated in CD141^+^DCs (p<0.01; **Figure 5F**), while an increase in the triple-positive CD40^+^ CD80^+^ CD86^+^ population was observed in CD1c^+^DCs (**Figure 5F**).

## Discussion

In this study we demonstrate that distinct inflammatory and chemotactic changes occur in circulating CD1c^+^DCs in IA, resulting in an accumulation of mature CD1c^+^DCs in both synovial tissue and SF. Synovial tissue CD1c^+^DCs are more mature than CD1c^+^DCs residing in peripheral blood or indeed healthy synovial tissue. Transcriptomic analysis of synovial CD1c^+^DCs identified an enrichment in genes associated with multiple inflammatory pathways including DC maturation, while SPICE analysis demonstrated the presence of unique co-expression maturation profiles that are absent in circulatory CD1c^+^DCs. Synovial CD1c^+^DCs are functionally mature and more responsive to TLR7/8 ligand stimulation while also exhibiting decreased antigen uptake and dysregulated MMP production. Finally, we report a direct comparison of synovial CD1c^+^DCs and CD141^+^DCs, where we identified distinct transcriptional variation in addition to differential expression of maturation markers that distinguish CD1c^+^DCs and CD141^+^DCs. These data suggest that synovial DCs reside in the synovium in distinct poly-maturational states, which may contribute to differential inflammatory effects within the synovium.

We report a significant decrease in circulating CD1c^+^DCs in IA peripheral blood compared to HC, concomitant with an increase in CD80, CD40, CXCR3, and CCR7. This is in agreement with a previous study demonstrating a decrease in circulating CD1c^+^DCs in early RA, which displayed increased expression of CCR7 and CD86 compared to HC (11). Moreover, transcriptomic analysis demonstrated an enrichment of genes associated with DC maturation, TLR signaling, and IL-1 signaling in IA circulatory and synovial CD1c^+^DCs compared to HC. Furthermore, we report a specific induction in genes involved in maturation and migration, both of which are key pathways involved in IA pathogenesis (6, 19–21). Collectively, this suggests that circulating IA CD1c^+^DCs are more mature, with increased inflammatory potential which migrate and accumulate in the IA synovium. In support of this hypothesis, single-cell analysis of synovial tissue demonstrated significant enrichment in CD1c^+^DCs in IA synovial tissue compared to peripheral blood and healthy synovial tissue. Previous studies utilizing histological techniques reported the presence of CD1c^+^ cells throughout the synovial sublining in RA and PsA (22); however, CD1c expression has also been reported on other APC including monocytes and B cells (23, 24). Therefore, our synovial single-cell analysis, with strict inclusion and exclusion gating criteria, demonstrates definitively the accumulation of bona fide CD1c^+^DCs in IA synovial tissue.

In addition to IA synovial tissue, we also report an accumulation of CD1c^+^DCs in IA SF compared to peripheral blood, which is in agreement with previous studies that demonstrated enrichment of DCs in SF (12, 13, 25). Consistent with our data, previous studies have demonstrated a mature CD83^+^DCs population expressing high levels of CCR7 localized to the synovial perivascular region in RA (15). Furthermore, an accumulation of CD33^+^CD14^−^ cells with increased NFκB activation has also been reported in RA synovial tissue (26). Interestingly, Segura et al. identified a novel mature inflammatory DCs population (CD1c^+^CD14^+^DCs) in RA SF, which is distinct from other previously described mDC populations (27).

Hierarchical clustering and PCA analysis revealed that synovial CD1c^+^DCs are transcriptionally distinct from circulatory CD1c^+^DCs consistent with our previous study demonstrating distinct transcriptional signatures in joint specific CD141^+^DCs (6), in addition to studies of DCs in other inflammatory sites (27). This suggests that the unique synovial microenvironment may imprint site-specific transcriptional changes onto newly recruited DCs. IPA also revealed multiple pathways enriched in synovial CD1c^+^DCs including TLR, mTOR, TREM-1, and STAT3 signaling, all of which have previously been implicated in the pathogenesis of IA in cells such as DCs, T cells, and synovial fibroblasts (6, 18, 28–30). Moreover, in agreement with our *ex-vivo* data, an enrichment of genes associated with DC maturation was also demonstrated in synovial CD1c^+^DCs compared to peripheral blood.

In this study, we demonstrate an increase in the expression of CD80, CD83, and CD40 in IA SF CD1c^+^DCs compared to peripheral blood in addition to significant increases in coinhibitory receptors PD-L1 and BTLA. Interestingly, synovial CD1c^+^DCs also express multiple costimulatory and coinhibitory markers simultaneously, indicative that multiple maturation states exist within the synovium. This is the first study to report poly-maturational states in synovial CD1c^+^DCs with previous studies reporting an increase in single-positive cells in IA. Soluble CD80, CD86, and CD83 were all reported to be increased in RA SF compared to serum (31), while SF DCs have higher expression of CD80, CD83, and CD86 compared with circulatory DCs (25). While the high expression of co-inhibitory markers PD-L1 and BTLA on mature synovial DCs may appear counterintuitive, we and others previously reported the upregulation of PD-1 and PD-L1 within the RA synovium (32–34), with multiple studies suggesting this pathway itself may be dysfunctional, leading to the unabated accumulation of self-reactive T cells in the joint (32, 33). The novel identification of unique synovial poly-maturational DCs highlights the complexity and spectrum of DC phenotypes in the joint and raises the question as to whether unique DC maturation subsets have overlapping or indeed divergent functional effects within the synovium.

Next, we demonstrate that synovial CD1c^+^DCs remain responsive to external stimuli as observed by increased expression of CD80, CD40, and CD86 following TLR7/8 stimulation. Given that CD1c^+^DCs express high levels of TLR7/8 and endogenous TLR7 ligands have previously been identified in RA SF, stimulation *via* the TLR7/8 receptor represents an effective model to examine if synovial DCs can respond to their environment despite their heightened maturation status (35, 36). Previous studies by Jongbloed et al. are in agreement with our observations whereby mature synovial mDCs stimulated *via* the TLR2 have heightened expression of CD40, CD80, and CD83, further suggestive that despite the mature nature of synovial DCs, they remain capable of further maturation (25).

A key immunological function of DCs, which remains intrinsically linked to their maturation status, is their ability to process antigen (37). Our data identify a potential functional consequence of the CD40L maturation status identified in synovial DCs, whereby synovial CD1c^+^DCs may have a decreased ability to process antigen compared to circulatory CD1c^+^DCs. In agreement with our data, Wilton et al. demonstrated that SF and peripheral blood polymorphonuclear (PMN) cells in RA have a decreased capacity to phagocytose *Candida albicans* (38). We also report dysregulated production of MMPs in synovial DCs compared to peripheral blood DCs with decreased production of MMP-1 with a concomitant trending increase in MMP-3 observed, both of which have been strongly implicated in the pathogenesis of IA (33, 35). Previous studies have also demonstrated increased levels of MMP-3 from mDCs from MS patients compared to controls. Moreover, cleavage by MMP-1 or MMP-3 can inactivate chemokines such as MCP-1, MCP-2, and MCP-4, thus resulting in the retention of cells within an inflamed site (39–42).

We next examined the transcriptomic and maturation profiles of two synovial mDCs, specifically CD141^+^DCs *vs* CD1c^+^DCs subsets to identify overlapping or distinct phenotypes that tissue-specific DCs may possess. PCA and hierarchical clustering analysis revealed distinct transcriptional variation between CD1c^+^DCs and CD141^+^DCs in addition to a predicted upregulation in pathways including chemokine, mTOR, and cdc42 signaling. Furthermore, we identified altered and opposing expression of several DC maturation markers between CD1c^+^DCs and CD141^+^DCs, with CD1c^+^ DCs expressing higher levels of CD86 and CD80, while CD141^+^DCs expressed higher levels of CD40 and BTLA indicating differential maturation patterns in each DC subset. CD1c+DC, with higher CD80 and CD86 expression, may be potentially more inflammatory than their synovial CD141^+^DC counterparts, which have expression of the inhibitory receptor BTLA. Moreover, CD141^+^DCs express high levels of Clec9A, which is known to bind to damaged cells *via* exposed F-actin. Therefore, we hypothesize that CD141^+^DCs may be recruited to the joint in response to ongoing synovial damage and thus possess an immunoregulatory function. Furthermore, previous studies have demonstrated that loss of CD28 on T cells results in defective Th2 responses, while a loss in CD40L contributes to defective Th1 development, suggestive that synovial CD1c^+^DC may induce more potent Th2 type responses while synovial CD141^+^DC may induce a stronger Th1 type response. Finally, using SPICE analysis, we determined that both synovial subsets have differential co-expression patterns of both costimulatory and co-inhibitory markers (specifically CD86, CD80, CD40, CD83, PD-L1, BTLA). This study directly compares synovial CD1c^+^DCs and CD141^+^DCs thus demonstrating that they express overlapping and distinct maturation markers and reside in the inflamed synovium in a poly-maturational state. While outside the scope of this study, it remains unknown, however, whether these distinct differences also occur in HC synovium. While maturation and transcriptional differences are likely to occur between the two subsets in the absence of inflammation, our data and results from previous studies (18) highlight the proinflammatory effect the synovial microenvironment has on DC function, thus suggesting that the distinct differences between CD1c^+^DC and CD141^+^DC may be enhanced within the inflamed synovium.

The limitations of this study include the difficulty in performing in-depth functional assays on synovial DCs. While DC populations are enriched within the synovium, the large numbers required to perform additional functional assays are outside the scope of this study. However, these studies characterize the frequency, activation, transcriptional analysis, and phagocytosis of DC subsets in the synovial compartment, in synovial tissue and fluid, with the majority of previous studies focused on blood. Based on our data, these two compartments differ in activation status, and therefore we believe this study significantly adds to the current knowledge in the field. Recent advances in single-cell technologies may enable more in-depth functional analysis of synovial DCs in the future. Furthermore, an additional limitation within this study is the small sample size used for RNA sequencing experiments. This is in part due to the large amount of synovial fluid that is needed to yield enough DC to perform bulk RNAseq. However, the gene expression profiles identified in both CD1c^+^DCs and CD141^+^DCs are strongly replicated across all three patients, suggesting that results obtained in this study have strong potential to be reproduced in larger studies in the future. Furthermore, while our data concur with previous studies examining the frequency of CD1c+DC in IA peripheral blood compared to HC (25), this work demonstrates the differential transcriptomic and phenotypic profiles of synovial *versus* peripheral blood CD1c^+^DCs. These data emphasize that examination of immune cell populations within the periphery does not always mirror the immune landscape that is present within the synovium, further emphasizing the need for studies such as this to characterize and highlight the differences that occur in DCs between the two sites. Furthermore, within this study we examined DCs in IA, thus including patients diagnosed with RA or PsA. Therefore, future work should aim at delineating potential phenotypic, maturational, or functional differences that may occur between DC in RA compared to other types of spondyloarthropathies.

Interestingly, recent studies have also broadened our understanding of DC in both the periphery and human inflamed tissues with the identification of DC3. While outside the scope of this study, it remains unknown what role DC3 may have in IA pathology either in the periphery or indeed the synovium itself. Future studies should aim at exploring the DC3 frequency and phenotype of DC3 in IA using high-dimensional techniques such as scRNAseq and extensive flow cytometric analysis.

In conclusion, this study demonstrates that peripheral blood CD1c^+^DCs in IA patients accumulate in the IA synovium, where they reside in a mature state. CD1c^+^DCs display unique co-expression patterns of maturation markers simultaneously, indicating that synovial DCs exist in multiple maturation states. Functionally, synovial CD1c^+^DCs are responsive to external stimuli, are less phagocytic, and have altered expression of MMPs compared to their blood counterparts. Finally, synovial CD1c^+^DCs and synovial CD141^+^DCs are transcriptionally distinct and express differential maturation markers. Collectively, this suggests that the specific targeting of one or more costimulatory markers may prove ineffective in dampening DC immune responses and instead therapeutic interventions should focus on preventing the differentiation of DCs along this poly-maturational pathway.

## Data Availability Statement

The datasets presented in this study can be found in online repositories. The names of the repository/repositories and accession number(s) can be found below: https://www.ncbi.nlm.nih.gov/, GSE151897; https://www.ncbi.nlm.nih.gov/, GSE108174.

## Ethics Statement

Ethical approval to conduct this study was granted by St. Vincent’s Healthcare Group Medical Research and Ethics Committee and the Tallaght Hospital/St. James’s Hospital Joint Research Ethics Committee, and all patients and healthy subjects gave fully informed written consent prior to inclusion. The patients/participants provided their written informed consent to participate in this study.

## Author Contributions

MC and UF conceived the experimental approach and designed the experiments. MC, VM, VB, SN, and UF performed the experiments, analyzed the data, and prepared the manuscript. DV recruited patients, analyzed the data, and prepared the manuscript. CH, RM, and PG recruited patients and obtained biosamples. UF supervised the project. All authors contributed to the writing of the article and approved the submitted version.

## Funding

This work was supported by the American Association of Immunologists, the Centre for Arthritis and Rheumatic Diseases, and Arthritis Ireland.

## Conflict of Interest

VP and SN were employed by Janssen Research & Development.

The remaining authors declare that the research was conducted in the absence of any commercial or financial relationships that could be construed as a potential conflict of interest.

## Publisher’s Note

All claims expressed in this article are solely those of the authors and do not necessarily represent those of their affiliated organizations, or those of the publisher, the editors and the reviewers. Any product that may be evaluated in this article, or claim that may be made by its manufacturer, is not guaranteed or endorsed by the publisher.

## Supplementary Material

Supplementary Figure 1 Gating strategy to identify CD1c^+^ DCs in PBMC, synovial tissue, and synovial fluid.

Supplementary Figure 2 Hierarchical clustering analysis of IA and HC peripheral blood CD1c +DCs RNA sequencing was performed on IA and HC PB CD1c+ DCs (n=3). Hierarchical clustering analysis on DEGs.

Supplementary Figure 3 Production of MMP-1, MMP-3, and MMP-9 from CD1c^+^ DCs from IA PBMC and IA SFMC (n = 4).
