# Supplementary figures and images for: Functionally Mature CD1c+ Dendritic Cells Preferentially Accumulate in the Inflammatory Arthritis Synovium
Source: Front Immunol. 2021 Oct 7;12:745226. doi: 10.3389/fimmu.2021.745226 (PMC8529992; doi:10.3389/fimmu.2021.745226)

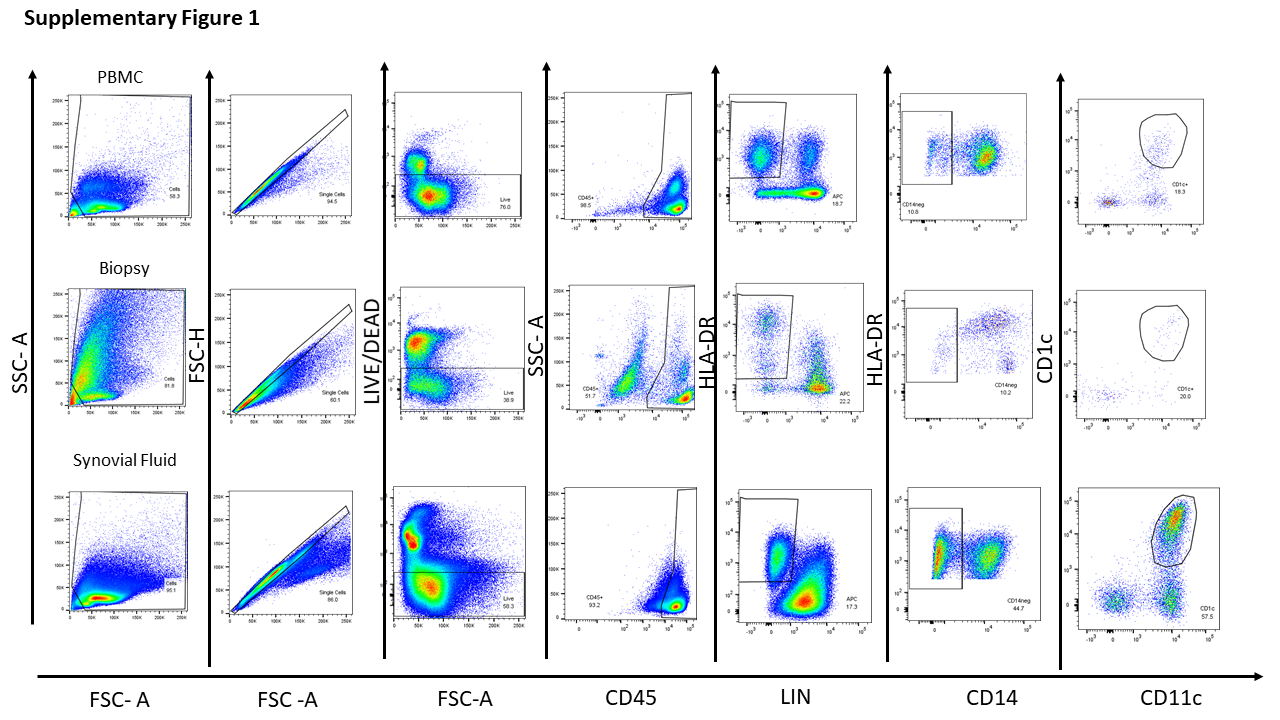

Supplement: Supplementary Figure 1 — Gating strategy to identify CD1c+ DCs in PBMC, synovial tissue, and synovial fluid. [file Image_1.tif]

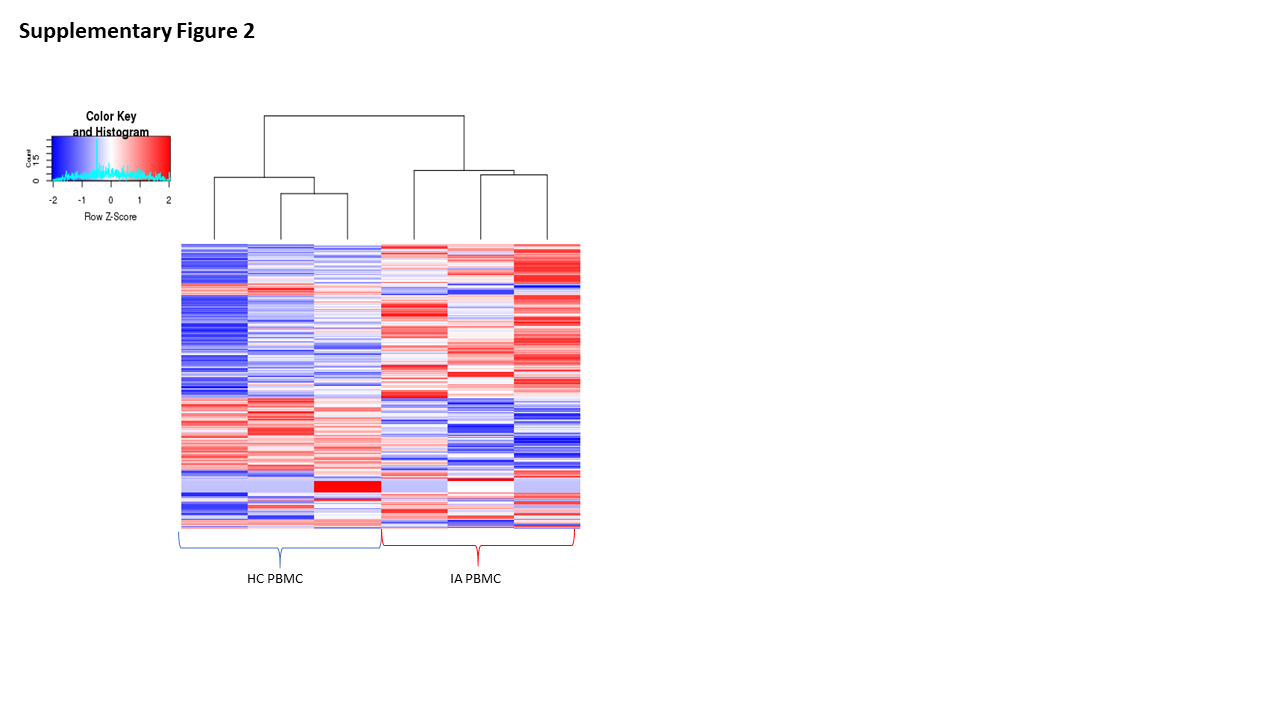

Supplement: Supplementary Figure 2 — Hierarchical clustering analysis of IA and HC peripheral blood CD1c +DCs RNA sequencing was performed on IA and HC PB CD1c+ DCs (n=3). Hierarchical clustering analysis on DEGs. [file Image_2.tif]

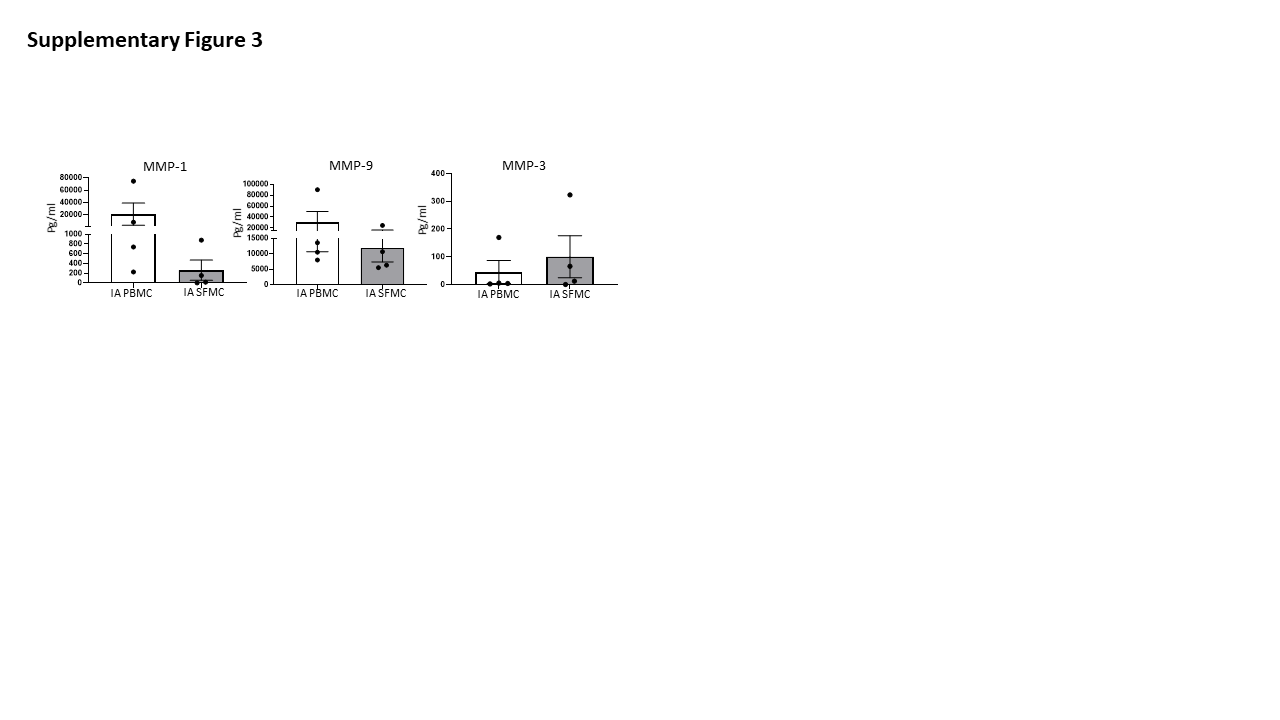

Supplement: Supplementary Figure 3 — Production of MMP-1, MMP-3, and MMP-9 from CD1c+ DCs from IA PBMC and IA SFMC (n = 4). [file Image_3.tif]
