# Supplementary material for: Functionally Mature CD1c+ Dendritic Cells Preferentially Accumulate in the Inflammatory Arthritis Synovium
Source: Front Immunol. 2021 Oct 7;12:745226. doi: 10.3389/fimmu.2021.745226 (PMC8529992; doi:10.3389/fimmu.2021.745226)
Supplement: Supplementary file 4 [file Table_1.docx]

| **PARAMETERS** | **RHEUMATOID ARTHRITIS (n=32)** | **PSORIATIC ARTHRITIS**  **(n=15)** |
| --- | --- | --- |
| **Gender :** Female | 57% | 57% |
| Male | 43% | 43% |
| **Treatment**: No medication | 35% | 21% |
| Methotrexate alone | 24% | 14% |
| Methotrexate in Combination | 17% | 7% |
| Biologics | 4% | 21% |
| Other | 10% | 21% |
| **Synovitis (**MEAN +/- SD) | 75.6 (+/- 11.4) | 74(+/- 13.9) |
| **DAS28** (MEAN +/- SD) | 3.7 (+/- 1.2) | 3.6 (+/- 0.8) |
