# Supplementary material for: Functionally Mature CD1c+ Dendritic Cells Preferentially Accumulate in the Inflammatory Arthritis Synovium
Source: Front Immunol. 2021 Oct 7;12:745226. doi: 10.3389/fimmu.2021.745226 (PMC8529992; doi:10.3389/fimmu.2021.745226)
Supplement: Supplementary file 5 [file Table_2.docx]

Supplementary Table 2: Flow Cytometry Antibodies

| Name | Fluorochrome | Clone | Company |
| --- | --- | --- | --- |
| Lineage | APC | UCHT1:HCD14;3G8;  HIB19;2H7;HCD56 | Biolegend |
| Lineage | FITC | 145-2C11;M1/70;RA3-  6B2;TER-119;RB6-8C5 | BD Biosciences |
| HLADR | V450 | L243 | Biolegend |
| CD11c | PerCp Cy5.5 | Bu15 | Biolegend |
| CD1c | Pe-Cy7 | L161 | ebioscience |
| CD40 | BV605 | 5C3 | Biolegend |
| CD40 | APCCy7 | 5C3 | Biolegend |
| CD80 | AF700 | L307.4 | BD Biosciences |
| CD80 | PE | L307.4 | BD Biosciences |
| CXCR3 | BV650 | G025H7 | Biolegend |
| CCR7 | PE/Dazzle 594 | G043H7 | Biolegend |
| CD45 | FITC | HI30 | BD Biosciences |
| CD83 | APC | HB15e | Bd Biosciences |
| PD-L1 | BV650 | 29E2A3 | Biolegend |
| BTLA | PECF594 | J168-540 | BD Biosciences |
| CD86 | Pe-Cy5 | IT2.2 | Biolegend |
| CD86 | FITC | 2331 | BD Biosciences |
| CD141 | BV510 | IA4 | BD Biosciences |
